# Two Chalcone Synthase Isozymes Participate Redundantly in UV-Induced Sakuranetin Synthesis in Rice

**DOI:** 10.3390/ijms21113777

**Published:** 2020-05-27

**Authors:** Hye Lin Park, Youngchul Yoo, Seong Hee Bhoo, Tae-Hoon Lee, Sang-Won Lee, Man-Ho Cho

**Affiliations:** 1 Graduate School of Biotechnology and Department of Genetic Engineering, Kyung Hee University, Yongin 17104, Korea; hlpark@khu.ac.kr (H.-L.P.); yooyc@khu.ac.kr (Y.Y.); shbhoo@khu.ac.kr (S.H.B.); 2 Department of Applied Chemistry, Kyung Hee University, Yongin 17104, Korea; thlee@khu.ac.kr

**Keywords:** chalcone synthase, sakuranetin, rice, sakuranetin biosynthesis, phytoalexin, UV

## Abstract

Chalcone synthase (CHS) is a key enzyme in the flavonoid pathway, participating in the production of phenolic phytoalexins. The rice genome contains 31 *CHS* family genes (*OsCHS*s). The molecular characterization of *OsCHS*s suggests that *OsCHS8* and *OsCHS24* belong in the bona fide CHSs, while the other members are categorized in the non-CHS group of type III polyketide synthases (PKSs). Biochemical analyses of recombinant OsCHSs also showed that OsCHS24 and OsCHS8 catalyze the formation of naringenin chalcone from *p*-coumaroyl-CoA and malonyl-CoA, while the other OsCHSs had no detectable CHS activity. OsCHS24 is kinetically more efficient than OsCHS8. Of the *OsCHS*s, *OsCHS24* also showed the highest expression levels in different tissues and developmental stages, suggesting that it is the major CHS isoform in rice. In *oschs24* mutant leaves, sakuranetin content decreased to 64.6% and 80.2% of those in wild-type leaves at 2 and 4 days after UV irradiation, respectively, even though *OsCHS24* expression was mostly suppressed. Instead, the *OsCHS8* expression was markedly increased in the *oschs24* mutant under UV stress conditions compared to that in the wild-type, which likely supports the UV-induced production of sakuranetin in *oschs24*. These results suggest that OsCHS24 acts as the main CHS isozyme and OsCHS8 redundantly contributes to the UV-induced production of sakuranetin in rice leaves.

## 1. Introduction

Phytoalexins are antimicrobial secondary metabolites, and their production is induced by pathogen infections and environmental stress [1]. Rice produces a variety of diterpenoid and phenolic phytoalexins in response to pathogen attacks as well as UV stress [2,3,4,5,6,7]. The flavonoid sakuranetin is a well-known phenolic phytoalexin in rice, which is the 7-*O*-methylated form of naringenin [3,6,7]. Sakuranetin was first isolated from UV-irradiated rice leaves, and was also detected in blast-infected rice leaves [3]. Naringenin *O*-methyltransferase (OsNOMT) for sakuranetin synthesis was purified from the UV-treated rice *oscomt1* mutant and the corresponding gene was identified [8]. The expression of *OsNOMT* was found to be induced in response to UV irradiation prior to sakuranetin accumulation [5]. Microarray and phytochemical analyses of UV-treated rice leaves revealed that the expressions of phenylpropanoid and flavonoid pathway genes including CHS and chalcone isomerase (CHI) genes are induced by UV and participate in sakuranetin production [5,6,7].

CHS is the first committed enzyme in the flavonoid pathway, which catalyzes the formation of naringenin chalcone from one *p*-coumaroyl-CoA and three malonyl-CoAs [9,10,11]. Naringenin chalcone is converted to naringenin by CHI to provide C_6_-C_3_-C_6_ backbones for a wide range of flavonoids [12]. The CHS superfamily is known as the plant type III PKS superfamily [9,10,13]. In addition to CHS, plant type III PKSs include diverse biosynthetic enzymes—such as stilbene synthase (STS), curcumin synthase, acridone synthase, bibenzyl synthase, and benzophenone synthase—providing backbones for a variety of plant secondary metabolites [9,10]. Type III PKSs are homodimeric enzymes of two identical subunits and have the Cys-His-Asn catalytic triad at the active site [9,10]. The crystal structures of CHSs, including *Medicago sativa* CHS2 (MsCHS2), reveal that CHS retains catalytic Cys-His-Asn residues [14,15].

The number of *CHS* family genes is highly variable among plant species. Eight copies of *CHS* genes were identified in bread wheat (*Triticum aestivum*) [16]. In maize and soybean, the *CHS* family consists of 14 genes [17,18]. A moss, *Physcomitrella patens*, contains 17 *CHS* members [19]. *Citrus* species were found to have 77 *CHS* family genes [20]. Homology searches of databases using the MsCHS2 sequence as a query have shown that the rice genome has more than 27 *CHS* family genes [21,22]. Several studies have reported the biochemical functions of rice *CHS* family genes rather than CHS, such as curcuminoid synthase (CUS) and alkylresorcylic acid synthase (ARAS) [21,23,24].

In the present study, we performed the molecular and biochemical characterization of *OsCHS*s and found that *OsCHS8* and *OsCHS24* encode the functional CHSs in rice. Analyses of sakuranetin accumulation and the expressions of *OsCHS24* and *OsCHS8* in a UV-treated *oschs24* mutant revealed their roles in sakuranetin biosynthesis under UV stress conditions.

## 2. Results

### 2.1. The Rice CHS Family

A search of the MSU RGAP Database revealed 31 genes that were annotated as putative CHSs and/or STSs, which together comprise the *CHS* family in rice (Table 1). This includes 27 previously identified *OsPKS*s [22]. CHS is a homodimer of two 40–45 kDa polypeptides [10,11]. Theoretical molecular masses of most OsCHSs were comparable with those of the functional CHSs. *OsCHS26–28* and *31* have very short open reading fames (ORFs) of 417–636 base pairs (bp) (Table 1). The ORFs of *OsCHS5*, *19*, *20*, and *21* were also significantly shorter than the typical lengths of *CHS*s, leading to a large deletion in the N-terminal region of CHS (Figure S1). Therefore, these *OsCHS*s are unlikely to encode functional CHS family enzymes. In contrast, *OsCHS11* has a long ORF of 1365 bp encoding a protein of 49.8 kDa, leading to an insertion of 50 amino acids in the middle of the N-terminal region of CHS (Figure S1).

**Figure 1:**
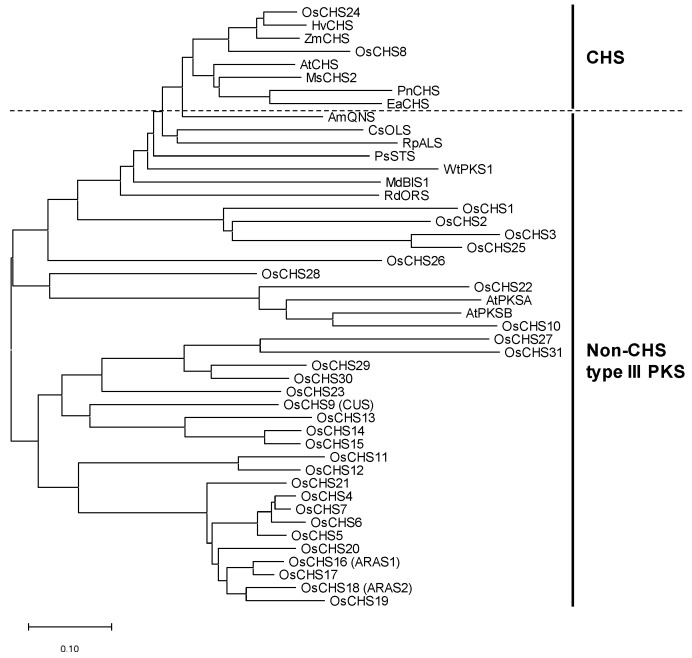
Phylogenetic tree of OsCHSs and other plant CHS family members. The amino acid sequences were aligned with Clustal-W and the neighbor-joining tree was built with MEGA ver. 6. Amino acid sequences used were AtCHS (AAB35812), ZmCHS (NP_001142246.1) EaCHS (Q9MBB1), HvCHS (CAA41250), MsCHS2 (P30074), PnCHS (BAA87922), AmQNS (AGE44110), CsOLS (B1Q2B6), RpALS (AAS87170), PsSTS (CAA43165), WtPKS1 (AAW50921), MdBIS1 (NP_001315967), RdORS (BAV83003), AtPKSA (O23674), and AtPKSB (Q8LDM2). QNS, OLS, ALS, BIS, and ORS stand for quinolone synthase, olivetol synthase, aloesone synthase, 3,5-dihydroxybiphenol synthase, and orcinol synthase, respectively.

Multiple alignments of the amino acid sequences of OsCHSs and other plant CHSs showed that OsCHS8 and OsCHS24 are highly homologous to other CHSs, showing similarities of 71–94% (Table S1). Phylogenetic analysis also indicated that OsCHS8 and OsCHS24 were closely related to the bona fide CHSs (Figure 1). The other OsCHSs showed similarities of 17–66% to CHSs (Table S1) and were categorized into the non-CHS group of type III PKSs (Figure 1), suggesting that they likely play different metabolic roles than CHS. OsCHS9, OsCHS16, and OsCHS18 showed 55–62% similarities to CHSs (Table S1). OsCHS9 was previously identified as CUS [23]. *OsCHS16* and *OsCHS18* were demonstrated to encode ARASs [21]. Phylogenetic analysis revealed that OsCHS10 and OsCHS22 are closely related to AtPKSA and AtPKSB, which are involved in the formation of the outer pollen wall (Figure 1) [25,26].

### 2.2. Analyses of the Conserved Residues and Motifs in the CHS Family

**Figure 2:**
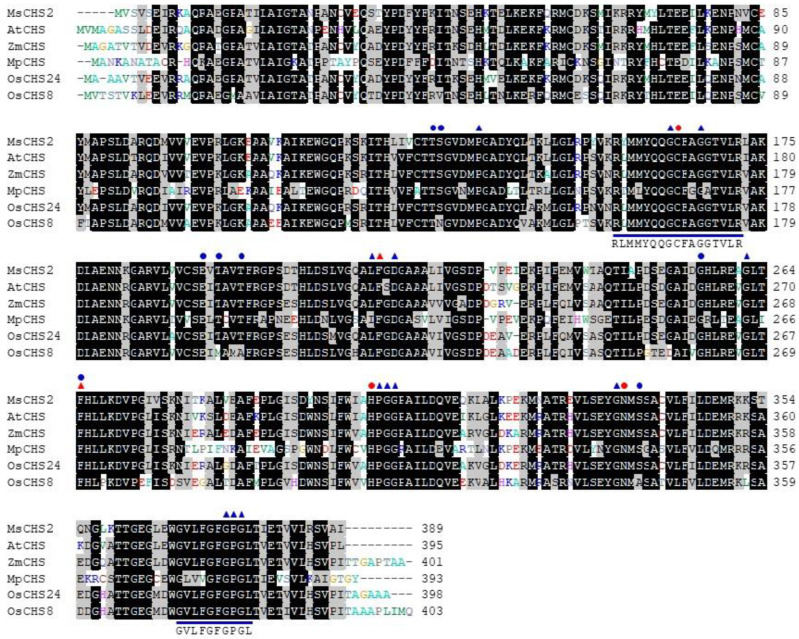
Multiple alignments of amino acid sequences of OsCHSs and other plant CHSs. The amino acid sequences were aligned with Clustal-W. Amino acid residues that are identical or similar are shaded in black and gray, respectively. Red circles indicate catalytic triad residues in CHSs. Residues marked with red triangles are gatekeeper Phe residues. Blue triangles are conserved residues shaping the active site geometry of CHS. The blue circles indicate residues important in the binding of the coumaroyl moiety and the specificity of cyclization reactions in CHSs. The conserved motifs in CHSs are underlined and their consensus sequences indicated. Amino acid sequences of other plant CHSs used were MsCHS2 (P30074), AtCHS (AAB35812), ZmCHS (NP_001142246), and MpCHS (CAD42328).

CHS contains three conserved residues, Cys^164^, His^303^, and Asn^336^ (all numbering of residues follows that of MsCHS2), which form a catalytic triad in the type III PKSs [9,10,14]. These catalytic residues are well conserved in most OsCHSs (Figure 2 and Figure S1). OsCHS19, 26, 27, and 31, however, lack more than one residue of the catalytic triad (Figure S1). Two Phe residues (Phe^215^ and Phe^265^) have been shown to be conserved in CHSs [9,10,14]. Phe^215^ is mostly conserved in OsCHSs except OsCHS27 that lacks the residue (Figure 2 and Figure S1). Phe^265^ is conserved in OsCHS8 and OsCHS24, whereas it is not strictly conserved in the non-CHS group of OsCHSs (Figure 2 and Figure S1). In addition to the catalytic triad, 13 residues shaping the geometry of the active site have been shown to be highly conserved in CHS enzymes [14,27]. These residues were absolutely conserved in about half of OsCHSs, including OsCHS8 and OsCHS24 (Figure 2 and Figure S1). Significant numbers of these residues are missing in OsCHS19, 26, 27 and 31. CHSs have highly conserved motifs of “RLMMYQQGCFAGGTVLR” and “GVLFGFGPGL” [28,29,30]. These motifs are absolutely conserved in OsCHS8 and OsCHS24, but vary in other OsCHSs (Figure 2 and Figure S1). In particular, OsCHS19, 26, and 31 were found to be missing one or both of these motifs. Based on the unusual ORF lengths and variations in the conserved residues and motifs, OsCHS5, 11, 19, 20, 21, 26, 27, and 31 were not expected to encode functional CHS family proteins.

### 2.3. Cloning and Heterologous Expression of OsCHSs

To identify the genes encoding functional CHS proteins, we attempted to clone *OsCHS*s other than the expected non-functional *OsCHS*s. The cDNAs of *OsCHS1–4*, *7–9*, *12*, *15*, *16*, *18*, *22*, *24*, and *25* were successfully cloned from rice leaves. Each *OsCHS* cDNA was inserted into the expression vector pET28a, and then the resulting constructs were transformed in to *Escherichia coli* BL21 (DE3). The recombinant OsCHS protein containing the N-terminal His-tag was produced in *E. coli* under various growth and induction conditions (Figure S2). OsCHS2, 7, 9, 12, 15, 16, 18, 22, 24, and 25 were successfully expressed as soluble forms in *E.coli* by 0.1 mM isopropyl β-D-thiogalactopyranoside (IPTG) under an induction temperature of 25 °C. OsCHS4 and 8 were produced in *E.coli* at 18 °C and at 0.1 mM IPTG concentration. OsCHS1 and 3 were produced only as insoluble forms under various growth temperatures and IPTG concentrations. The recombinant OsCHS proteins were purified with Ni^2+^ affinity chromatography (Figure S2).

### 2.4. CHS Activity and Kinetic Parameters of the Recombinant OsCHSs

**Figure 3:**
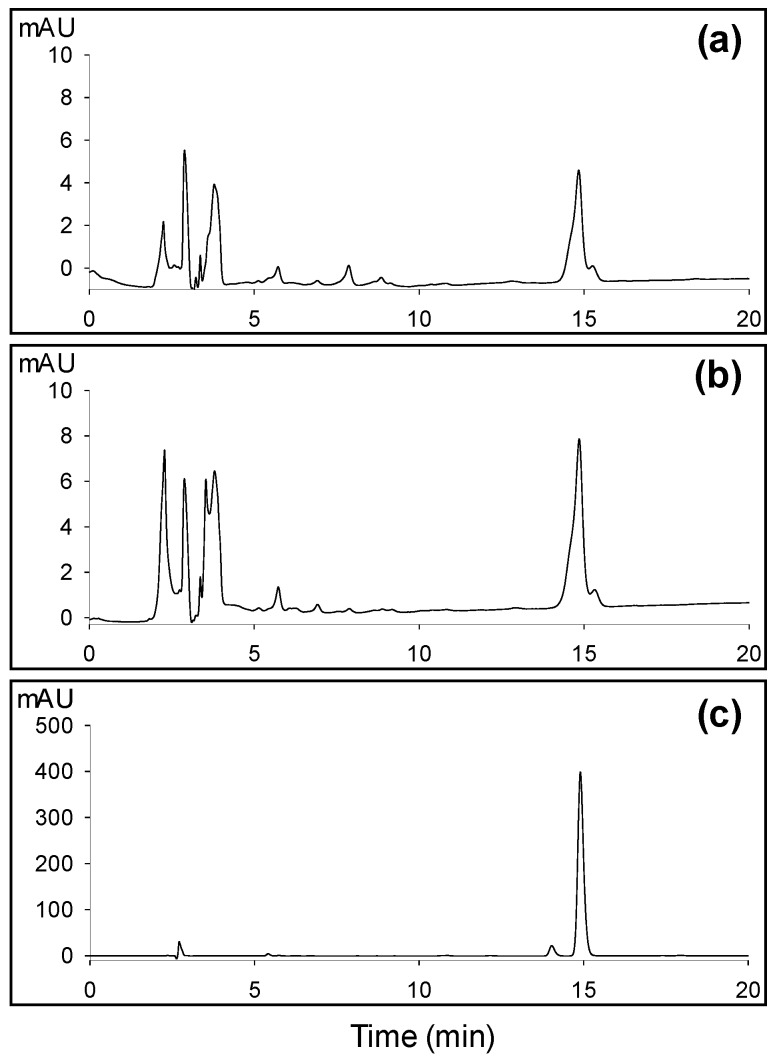
CHS activity assay of recombinant OsCHS8 (**a**) and OsCHS24 (**b**). The tetrahydroxychalcone was formed from *p*-coumaroyl-CoA and malonyl-CoA by OsCHS8 and OsCHS24. The resulting chalcone was spontaneously converted to naringenin, which was analyzed by reversed-phase HPLC. (**c**) HPLC chromatogram of the authentic naringenin.

The CHS activity of the recombinant OsCHS proteins was assayed with *p*-coumaroyl-CoA and malonyl-CoA as substrates. Naringenin chalcone synthesized by CHS is spontaneously converted to naringenin in aqueous solutions [31,32]. Therefore, the CHS activity of the recombinant OsCHSs was determined by analyzing the accumulation of naringenin using HPLC. In accordance with the strong conservation of the important residues of CHS activity, among 12 recombinant OsCHS proteins examined, OsCHS8 and OsCHS24 exhibited CHS activity (Figure 3 and Figure S3). All examined recombinant OsCHSs belonged in the non-CHS group showed no detectable CHS activity (Figure S3). This result suggests that among *OsCHS* family members, *OsCHS24* and *OsCHS8* encode biochemically functional CHS proteins.

The kinetic parameters of recombinant OsCHS8 and OsCHS24 were determined towards the *p*-coumaroyl- and malonyl-CoA substrates (Table 2). The *K*_M_ values of OsCHS24 and OsCHS8 for *p*-coumaroyl-CoA were 45.44 and 27.64 µM, respectively (Table 2). Both OsCHS24 and OsCHS8 showed similar affinity for malonyl-CoA, with *K*_M_ values of 47.42 and 59.38 µM, respectively. OsCHS24 showed a higher catalytic efficiency than OsCHS8, with *k*_cat_/*K*_M_ values of 1137.05 and 539.81 M^−1^ min^−1^, respectively.

### 2.5. In Silico and qRT-PCR Analyses of OsCHS Expression

**Figure 4:**
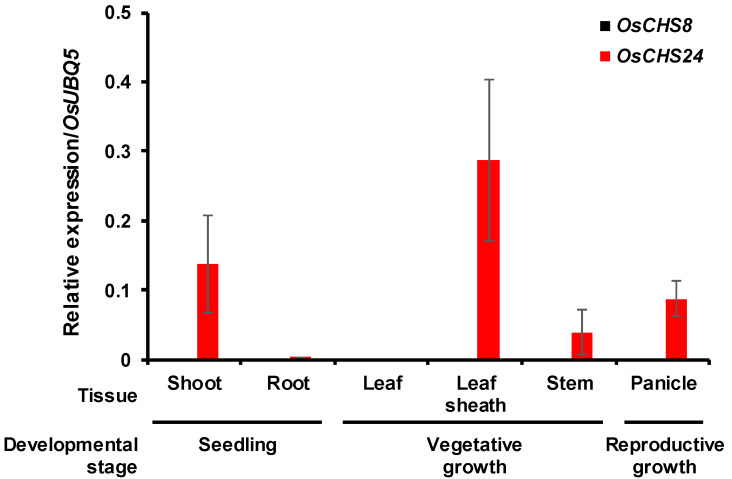
Quantitative real-time PCR analysis of *OsCHS24* and *OsCHS8* gene expression in rice seedlings and different adult tissues. Shoot and root samples were collected from seven-day-old rice seedlings. Leaf, leaf sheath, stem, and panicle samples were obtained from rice plants in vegetative and reproductive stages. An ubiquitin 5 gene (*OsUBQ5*) was used as an internal control. Expression levels of each *OsCHS* gene are presented as relative expression compared to *OsUBQ5* mRNA level. qRT-PCR analysis was performed on the triplicated biological samples. The results represent mean ± standard deviation.

The in silico expression analysis revealed that among *OsCHSs*, *OsCHS24* showed the highest expression levels at different developmental stages (Figure S4). The expression levels of the other *OsCHS*s were very low at all developmental stages (Figure S4). The quantitative real-time PCR (qRT-PCR) analysis also showed that *OsCHS24* is highly expressed in shoot tissues at an early growth stage and in the leaf sheath, stem, and panicle tissues in adult rice plants (Figure 4). Hu et al. [22] examined the *OsCHS* expressions in root, stem, leaf, young flower, and adult flower tissues and found that *OsCHS24* was expressed in the rice stem tissue. It was also reported that *OsCHS24* was expressed in rice seedlings [33]. The transgenic expression of *OsCHS24* was also shown to restore flavonoid accumulation in the *Arabidopsis* transparent *testa 4* mutant [33]. These findings suggest that *OsCHS24* acts as the major CHS isoform in rice. The expression of *OsCHS8*, another gene encoding a functional CHS, was very low at all examined developmental stages and tissues under normal growth conditions.

### 2.6. Sakuranetin Accumulation and *OsCHS24* and *OsCHS8* Expression in the UV-Irradiated *oschs24*

**Figure 5:**
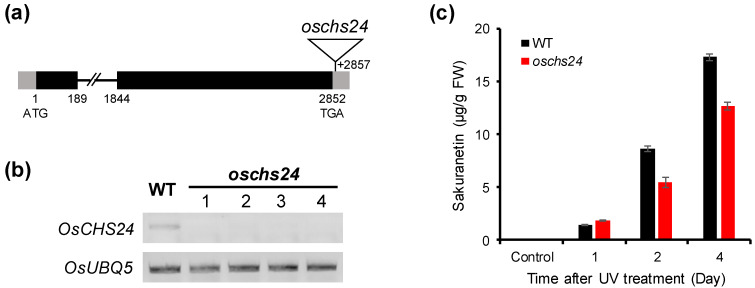
Characterization of the *oschs24* mutant. (**a**) The gene structure of *OsCHS24* showing the T-DNA insertion position of the *oschs24* mutant. Black boxes, gray boxes, and lines between boxes indicate exons, UTRs and introns, respectively. A triangle represents the position of the T-DNA insertion in *oschs24*. (**b**) RT-PCR analysis of *OsCHS24* expression in the leaves of homozygous *oschs24* mutants. The homozygous *oschs24* plants showed the suppressed expression of *OsCHS24*. *OsUBQ5* was used as an internal control. (**c**) Accumulation of sakuranetin in wild-type and *oschs24* mutant in response to UV irradiation. The leaf samples were collected from UV-treated rice plants 1, 2, and 4 days after UV irradiation. Analyses of the sakuranetin contents were performed on triplicate biological samples. The results represent the mean ± standard deviation. FW; fresh weight.

**Figure 6:**
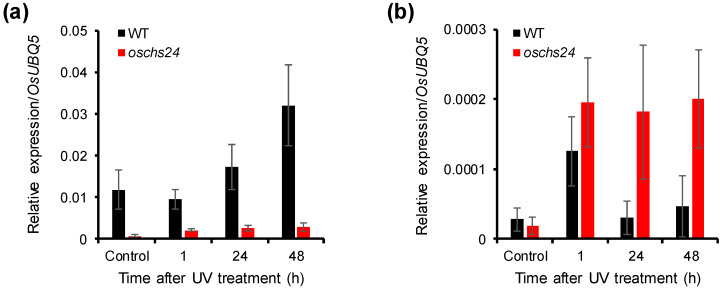
Analysis of the *OsCHS24* (**a**) and *OsCHS8* (**b**) expression in *oschs24* in response to UV irradiation. Transcript levels of *OsCHS24* and *OsCHS8* in the wild-type an *oschs24* mutants were analyzed by qRT-PCR. An ubiquitin 5 gene (*OsUBQ5*) was used as an internal control. Expression levels of each *OsCHS* gene are presented as relative expression compared to the *OsUBQ5* mRNA level. qRT-PCR analysis was performed on triplicate biological samples. The results represent mean ± standard deviation.

To ascertain the role of *OsCHS24* in sakuranetin accumulation, we characterized the *oschs24* mutant isolated from the rice T-DNA insertion mutant population [34]. The *oschs24* mutant has the T-DNA insertion in the 3’ UTR region of *OsCHS24* (Figure 5a). In homozygous *oschs24* mutants, the expression of *OsCHS24* was mostly suppressed by the insertion of T-DNA (Figure 5b). *OsCHS24* expression was increased in wild-type plants by UV irradiation, whereas it was not induced in UV-treated *oschs24* leaves (Figure 6a). The sakuranetin content in UV-treated leaves of *oschs24* were analyzed to elucidate the effect of the suppressed expression of *OsCHS24*. The sakuranetin content was not drastically decreased in UV-treated leaves of *oschs24* (Figure 5c), although the *OsCHS24* expression in *oschs24* was mostly suppressed even under UV stress conditions (Figure 6a). The sakuranetin content in *oschs24* was 64.6% and 80.2% of that in the wild-type at 2 and 4 days after UV irradiation, respectively (Figure 5c).

Unlike *OsCHS24*, the expression of *OsCHS8*, which encode another functional CHS isozyme, was immediately increased by UV irradiation. The induced expression levels were maintained for 48 h after UV irradiation (Figure 6b). The *OsCHS8* expression, however, reached its peak at 1 h after UV-treatment and then decreased to non-UV-treated levels in the wild-type (Figure 6b). The small decrease of sakuranetin contents and the maintained induction of *OsCHS8* in UV-treated *oschs24* leaves suggest that *OsCHS8* contributes to sakuranetin biosynthesis in *oschs24* under UV stress conditions.

## 3. Discussion

### 3.1. OsCHS24 and OsCHS8 Encode Functional CHSs in Rice

Although the *CHS* families are comprised of multiple genes, a few of them act as bona fide CHSs and the others participate in different metabolic processes [20,26,35]. *Arabidopsis* contains four *CHS* family genes, of which one gene (*AtCHS*) has been identified to participate in flavonoid biosynthesis [26,36]. Two *Arabidopsis CHS* family members (*AtPKSA* and *AtPKSB*) have been shown to encode hydroxyalkyl α-pyrone synthases, which are involved in the synthesis of sporopollenin, the major constituent of exine in the outer pollen wall [25,26]. In soybean, two *CHS* genes were found to be closely related to bona fide CHSs [18]. Of the *OsCHS*s, *OsCHS24* and *OsCHS8* were shown to be closely related to other bona fide CHSs (Figure 1 and Figure 2).

Each subunit of the CHS homodimer contains an independent active site catalyzing the elongation of the ketide chain on the *p*-coumaroyl starter molecule and cyclization of the tetraketide intermediate to form the chalcone [9,14]. In CHSs, two gatekeeper Phe residues (Phe^215^ and Phe^265^) positioned in the lower portion of the CoA-binding tunnel and the active site cavity were shown to facilitate substrate loading and the proper folding of the tetraketide intermediate in the cyclization reaction [9,10,14]. Along with Cys^164^, His^303^, and Asn^336^, Phe^215^ were known to be conserved in all CHSs and other type III PKSs [9,14]. Phe^265^ is important in substrate specificity and is conserved in CHSs, whereas it varies in other type III PKSs [9,10,37]. OsCHS9 includes the substitution of Phe^265^ with Gly and has no detectable CHS activity (Figures S1 and S3). Instead of CHS, *OsCHS9* was shown to encode CUS [23,24]. OsCHS24 and OsCHS8 contain both Phe residues and indeed, exhibited CHS activity. These findings suggest that *OsCHS24* and *OsCHS8* encode functional CHSs in rice (Figure 2 and Figure 3).

Divergent Type III PKSs differ in their preferences for starter molecules, degree of polyketide elongation, and intramolecular cyclization patterns. Subtle changes in the structure of their active sites lead to alterations in their kinetic properties and substrate/product specificities of type III PKSs [9,37,38]. Thr^132^, Ser^133^, Glu^192^, Thr^194^, Thr^197^, Gly^256^, Phe^265^, and Ser^338^ in CHS are important in the binding of the coumaroyl moiety and the cyclization reaction [9,10,14]. All of these residues are conserved in OsCHS24, and some of them are substituted in OsCHS8 (Figure 2). In OsCHS8, Ser^133^, Thr^194^, Thr^197^, and Ser^338^ are substituted for Asn, Met, Ala, and Ala, respectively (Figure 2), which likely leads to the lower catalytic efficiency of OsCHS8 compared to OsCHS24. In *Grewia asiatica*, two isoforms of CHSs, GaCHS1 and GaCHS2, were characterized to have CHS activity and showed different catalytic efficiencies towards *p*-coumaroyl-CoA [30]. Although important residues for the CHS function are highly conserved in both GaCHSs, Thr^132^ and Ser^133^ are substituted for His and Ala in GaCHS1, respectively, which may lead to differences in the kinetic properties of the isozymes. These residues have shown to be specifically substituted in other type III PKSs and are thought to define the substrate and product specificity of the enzyme [10,37]. The recombinant OsCHS9 protein was shown to have CUS activity catalyzing the formation of bisdemethoxycurcumin from two *p*-coumaroyl-CoAs and one malonyl-CoA [23,24]. In OsCHS9, Thr^132^, Thr^197^, Gly^256^, and Phe^265^ are replaced with Asn, Tyr, Met, and Gly, respectively (Figure S1), which enlarges the entrance and downward pocket of the CUS active site to accommodate the *p*-coumaroyldiketide intermediate and the second coumaroyl unit [24,37]. OsCHS16 and OsCHS18 were identified as ARAS1 and ARAS2, respectively, which catalyze the formation of alkylresorcylic acids from fatty acyl-CoAs and three malonyl-CoAs [21]. In OsCHS16 and OsCHS18, Thr^132^, Thr^197^, and Gly^256^ are substituted with Tyr, Cys, and Met, respectively (Figure S1). Thr^132^, Gly^256^, and Phe^265^ are conserved in both OsCHS24 and OsCHS8, whereas they are mostly substituted with other amino acids in OsCHSs of the non-CHS group—such as OsCHS9, OsCHS16, and OsCHS18 (Figure 2 and Figure S1)—suggesting that these residues are critical for the substrate and product specificity of CHSs.

### 3.2. OsCHS24 and OsCHS8 Redundantly Contribute to the UV-Induced Accumulation of Sakuranetin in Rice Leaves

As the first committed enzyme for flavonoid biosynthesis, CHS plays an important role in plant defense [11]. It has been demonstrated that the expressions of *CHS* genes are induced by diverse stresses, which leads to the production of defensive compounds including phytoalexins [11]. The constitutive expression in different developmental stages (Figure S4) and strong CHS activity (Table 2) of *OsCHS24* suggest that it is the major CHS isoform in rice. Along with UV-induced sakuranetin accumulation (Figure 5c), *OsCHS24* expression was stimulated in rice leaves in response to UV irradiation (Figure 6a). In a previous study, we suggested that the expression of *OsCHS24* was induced by UV and is relevant to sakuranetin accumulation in the UV-irradiated rice leaves [5,6]. The *oschs24* mutant was analyzed to confirm the role of *OsCHS24* in the UV-induced production of sakuranetin. Unexpectedly, the sakuranetin content was not significantly decreased in the UV-treated leaves of *oschs24*, despite the suppression of *OsCHS24* expression (Figure 5c).

Although *OsCHS8* encodes functional CHS, its expression levels were very low in different developmental stages and tissues (Figure 4 and Figure S4). Similarly, Shih et al. [33] reported that *OsCHS8* expression was not detected in rice seedlings. In wild-type rice leaves, *OsCHS8* expression was found to be induced immediately after UV irradiation and then decreased to the non-UV-treated level (Figure 6b). Interestingly, *OsCHS8* was more strongly induced in *oschs24* leaves than in wild-type leaves, and the induced transcript levels were maintained for 48 h after UV irradiation (Figure 6b), which likely complements the sakuranetin production in *oschs24* under UV stress conditions. This finding, along with a small decrease of the sakuranetin content in UV-treated *oschs24* leaves, suggests that *OsCHS24* acts as the main CHS isoform and *OsCHS8* contributes redundantly to the UV-induced production of sakuranetin in rice leaves.

## 4. Materials and Methods

### 4.1. Plant Growth, UV Treatment, and Materials

The seeds of *oschs24* mutant (line no. 2C-70073) and its Dongjin background rice (*Oryza sativa* L. spp. *Japonica* cv. *Dongjin*) were obtained from the rice T-DNA insertion mutant population in Crop Biotech Institute, Kyung Hee University, Korea [34]. The wild-type and the *oschs24* mutant seeds were sterilized and germinated on Murashige and Skoog medium (Duchefa, Harlem, The Netherlands) in a growth chamber at 28 °C. Seven-day-old seedlings were transferred to soil, and grown in a greenhouse for 6–7 weeks to analyze sakuranetin content. UV-treatment of rice plants was performed according to the methods described by Park et al. [5]. For the expression analysis of *OsCHS*s, root and shoot samples were collected from seven-day-old seedlings. Rice stem and leaf samples were collected from 10-week-old wild type rice plants, and panicles were collected from 14-week-old rice plants.

Malonyl-CoA was purchased from Sigma-Aldrich (St. Louis, MO, USA). *p*-Coumaroyl-CoA was synthesized by the method described by Beuerle and Pichersky [39]. Other reagents used in this study were purchased from Sigma-Aldrich, Thermo-Fisher Scientific (Waltham, MA, USA), Duchefa, and Samchun Chemicals (Seoul, Korea).

### 4.2. Multiple Sequence Alignment and Phylogenetic Analysis

Protein sequences of CHSs and other type III PKSs were obtained from the MSU RGAP database (http://rice.plantbiology.msu.edu/) [40] and the National Center for Biotechnological Information (https://www.ncbi.nlm.nih.gov/) database. The retrieved sequences were aligned using Clustal-W [41], and the phylogenetic tree was built by the neighbor-joining method using MEGA ver. 6 [42].

### 4.3. Cloning of OsCHSs

The first strand cDNA was synthesized using SuPrimeScript RT premix (GeNet Bio, Daejeon, Korea) with an oligo dT primer from total RNA extracted from eight-week-old rice leaves with RNAiso (Takara, Shiga, Japan). Cloning primers for *OsCHS*s and polymerase chain reaction (PCR) conditions are summarized in Table S2. *OsCHS*s were amplified from the first strand cDNA with Solg^TM^ Pfu DNA Polymerase (SolGent, Daejeon, Korea). The resulting PCR products were subcloned into the pGEM^TM^-T Easy vector (Promega, Madison, WI, USA) or the pJET 1.2 blunt cloning vector (Thermo-Fisher Scientific), and then their sequences were confirmed. Each *OsCHS* was cut with the appropriate restriction enzymes and inserted into the pET28a (+) vector (Novagen, Madison, WI, USA). The resulting *OsCHS*/pET28a(+) constructs were individually transformed into *E. coli* BL21(DE3) cells for heterologous expression of OsCHSs.

### 4.4. Expression and Purification of Recombinant OsCHSs

*E. coli* cells bearing the *OsCHS*/pET28a(+) construct were grown at 37 °C until an OD_600_ of ~0.6 in LB medium containing kanamycin (25 µg/mL). To induce the production of OsCHSs, 0.1 mM IPTG was added into the culture, followed by an additional incubation for 16 h at 18 or 25 °C. After induction, the cells were harvested by centrifugation (5000× *g* for 15 min). The harvested cells were resuspended in phosphate-buffered saline (PBS, 137 mM NaCl, 2.7 mM KCl, 10 mM Na_2_HPO_4_, 2 mM KH_2_PO_4_) supplemented with 1 mg/mL lysozyme and 1 mM phenylmethylsulfonyl fluoride. The cells were suspended in PBS and sonicated on ice, and then centrifuged at 15,900× *g* for 20 min at 4 °C. The crude extract was mixed with Ni-NTA agarose beads (Qiagen, Hilden, Germany) and incubated at 4 °C for 2 h with agitation. The mixtures were packed into a chromatography column and washed three times with a five-column volume of 20 mM imidazole in Tris buffer (50 mM Tris, pH 8.0, 300 mM NaCl). The recombinant OsCHSs were eluted with 50 to 100 mM imidazole in Tris buffer. The eluted proteins were analyzed by sodium dodecyl sulfate-polyacrylamide gel electrophoresis.

### 4.5. CHS Activity Assay and Steady-State Kinetics

OsCHS activity was measured according to the method of Zuubier et al. [43]. The enzyme activities of the purified recombinant OsCHSs were examined with *p*-coumaroyl- and malonyl-CoAs as substrates. The standard enzyme assay consisted of 80 µM *p*-coumaroyl-CoA and 160 µM malonyl-CoA in 0.1 M phosphate buffer (pH 7.2) with 30 µg of recombinant OsCHSs in total volume of 500 µL. The mixtures were incubated at 30 °C for 1h, and extracted twice with ethyl acetate. The extracts were evaporated, and the resulting residues were dissolved in methanol. The reaction products were analyzed using a reversed-phase high-performance liquid chromatography (HPLC) equipped with a Sunfire C_18_ column (Waters, Milford, MA, USA) following the elution and detection conditions described by Park et al., (2013). To determine the steady-state kinetic parameters of the CHS reactions, the *p*-coumaroyl-CoA and malonyl-CoA concentrations used were 5–100 µM and 10–100 µM, respectively. The assays were performed in triplicate and results represented as mean ± standard deviation.

### 4.6. In Silico and Quantitative Real-Time PCR Analysis of the OsCHS Gene Expression

Microarray data for *OsCHS*s at different development stages of rice were downloaded from the Genevestigator plant biology database (https://genevestigator.com/gv/doc/intro_plant.jsp) [44]. The normalized data were used to generate heatmap expression patterns using the Multi Experiment Viewer program (http://www.tm4.org/mev.html).

cDNAs synthesized from different tissues and developmental stages of rice were used as a template for qRT-PCR using a Prime Q-Mastermix (GeNet Bio, Daejeon, Korea) on an AriaMx real-time PCR system (Agilent, Santa Clara, CA, USA). Transcript levels were normalized by rice ubiquitin 5 (*OsUBQ5*) transcripts as a control. The ΔCt method was applied to calculate expression levels. We used primers that showed a single peak in melting curve data. The sequences and annealing temperatures of primers for qRT-PCR are listed in Table S3.

### 4.7. Analysis of Sakuranetin Content

Rice leaf samples collected from UV-treated and untreated rice plants were ground in liquid nitrogen and extracted with 70% methanol-water for 1 h with agitation. The aqueous methanol extracts were fractionated with ethyl acetate to enrich phenolic compounds. The ethyl acetate phases were dried in vacuo and the residues were dissolved in a small volume of methanol. The phenolic enriched fractions were used to measure sakuranetin contents using HPLC with the method described above.

## Figures and Tables

**Table 1:** Rice *CHS* family. The MSU RGAP Database search revealed 31 genes that were annotated as putative CHSs and/or STSs, which comprise the *CHS* family in rice.

Locus ID.	Name	Gene Description in the RGAP DB	ORF Length	Protein	Theoretical
			(bp)	Size (aa)	Mass (kDa)
Os01g41834	*OsCHS1*	Chalcone synthase, putative	1200	399	41.8
Os04g01354	*OsCHS2*	Chalcone synthase, putative	1182	393	42.7
Os04g23940	*OsCHS3*	Chalcone synthase, putative	1122	373	39.9
Os05g12180	*OsCHS4*	Chalcone synthase, putative	1179	392	42.6
Os05g12190	*OsCHS5*	Chalcone synthase, putative	939	312	333
Os05g12210	*OsCHS6*	Chalcone synthase, putative	1179	392	42.6
Os05g12240	*OsCHS7*	Chalcone synthase, putative	1179	392	42.7
Os07g11440	*OsCHS8*	Chalcone synthase, putative	1212	403	43.9
Os07g17010	*OsCHS9*	Chalcone synthase, putative	1209	402	43.2
Os07g22850	*OsCHS10*	Chalcone and stilbene synthase, putative	1290	429	46.5
Os07g31750	*OsCHS11*	Chalcone synthase, putative	1365	454	49.8
Os07g31770	*OsCHS12*	Chalcone synthase, putative	1218	405	42.8
Os07g34140	*OsCHS13*	Chalcone synthase, putative	1197	398	43
Os07g34190	*OsCHS14*	Chalcone and stilbene synthase, putative	1197	398	42.6
Os07g34260	*OsCHS15*	Chalcone and stilbene synthase, putative	1200	399	42.4
Os10g07040	*OsCHS16*	Chalcone synthase, putative	1197	398	43.2
Os10g07616	*OsCHS17*	Chalcone synthase, putative	1197	398	43.2
Os10g08620	*OsCHS18*	Chalcone and stilbene synthase, putative	1200	399	43.2
Os10g08670	*OsCHS19*	Chalcone synthase, putative	1032	343	37.5
Os10g08710	*OsCHS20*	Chalcone synthase, putative	885	294	31.6
Os10g09860	*OsCHS21*	Chalcone synthase, putative	1092	363	39.2
Os10g34360	*OsCHS22*	Stilbene synthase, putative	1170	389	42.2
Os11g32620	*OsCHS23*	Chalcone synthase, putative	1224	407	42.6
Os11g32650	*OsCHS24*	Chalcone synthase, putative	1197	398	43.4
Os11g35930	*OsCHS25*	Chalcone synthase, putative	1200	399	42.9
Os03g47000	*OsCHS26*	Chalcone synthase 1, putative	417	138	14.6
Os05g41645	*OsCHS27*	Chalcone synthase, putative	438	145	15.6
Os11g32540	*OsCHS28*	Chalcone synthase, putative	636	212	22.2
Os11g32580	*OsCHS29*	Chalcone synthase	1242	413	43.9
Os11g32610	*OsCHS30*	Chalcone and stilbene synthases, putative	1206	401	42.4
Os12g07690	*OsCHS31*	Chalcone synthase, putative	426	142	14.9

**Table 2:** Steady-state kinetic parameters of recombinant OsCHS24 and OsCHS8

OsCHS	*p*-Coumaroyl-CoA				Malonyl-CoA
	*K*_M_ (μM)	*V*_max_ (nmol min^−1^ mg^−1^)	*k*_cat_ (min^−1^)	*k*_cat_/*K*_M_ (M^−1^ min^−1^)	*K*_M_ (μM)
OsCHS8	27.64 ± 4.21	0.352 ± 0.02	0.0149	539.81	59.38 ± 13.83
OsCHS24	45.44 ± 2.94	1.218 ± 0.05	0.0517	1137.05	47.42 ± 8.21

## Supplementary Materials

Supplementary materials can be found at https://www.mdpi.com/1422-0067/21/11/3777/s1. Figure S1. Multiple alignments of amino acid sequences of OsCHSs and other plant CHSs. Figure S2. Purification of the recombinant OsCHSs heterologously expressed in *E. coli.* Figure S3. CHS activity assay of recombinant OsCHSs. Figure S4. In silico expression analysis of *OsCHS*s in different developmental stages of rice plants. Table S1. Amino acid sequence similarity percentage between the OsCHS family members and other plant CHSs. Table S2. Primer sequences and PCR conditions for *OsCHS* cloning. Table S3. Primer sequences for quantitative real-time PCR analysis.

## Abbreviations

CHS	chalcone synthase
PKS	polyketide synthase
OsCHS	rice chalcone synthase
OsNOMT	rice naringenin *O*-methyltransferase
CHI	chalcone isomerase
STS	stilbene synthase
MsCHS2	*Medicago sativa* chalcone synthase 2
CUS	curcuminoid synthase
ARAS	alkylresorcylic acid synthase
ORF	open reading frame
bp	base pair
IPTG	isopropyl β-D-thiogalactopyranoside
PBS	phosphate-buffered saline
qRT-PCR	quantitative real-time polymerase chain reaction
HPLC	high-performance liquid chromatography
OsUBQ5	rice ubiquitin 5
