# Supplementary material for: Two Chalcone Synthase Isozymes Participate Redundantly in UV-Induced Sakuranetin Synthesis in Rice
Source: Int J Mol Sci. 2020 May 27;21(11):3777. doi: 10.3390/ijms21113777 (PMC7312121; doi:10.3390/ijms21113777)
Supplement: Supplementary file 1 [file ijms-21-03777-s001.zip › ijms-802367-Supplementary-2/ijms-802367-supplementary-english done.docx]

**Table S1.** Amino acid sequence similarity percentage between the OsCHS family members and other plant CHSs

|  | **AtCHS** | **NtCHS** | **ZmCHS01** | **ZmCHS02** | **PhCHSA** | **FhCHS** | **GhCHS** | **EaCHS** | **HvCHS** | **MsCHS2** | **PsCHS** |
| --- | --- | --- | --- | --- | --- | --- | --- | --- | --- | --- | --- |
| OsCHS1  OsCHS2  OsCHS3  OsCHS4  OsCHS5  OsCHS6  OsCHS7  OsCHS8  OsCHS9  OsCHS10  OsCHS11  OsCHS12  OsCHS13  OsCHS14  OsCHS15  OsCHS16  OsCHS17  OsCHS18  OsCHS19  OsCHS20  OsCHS21  OsCHS22  OsCHS23  OsCHS24  OsCHS25  OsCHS26  OsCHS27  OsCHS28  OsCHS29  OsCHS30  OsCHS31 | 59  65  59  58  49  58  60  82  60  49  51  59  57  59  58  59  60  60  50  45  55  52  59  89  64  23  19  35  55  56  18 | 54  60  53  55  44  54  56  77  58  45  50  56  56  56  55  56  56  56  47  43  50  48  55  84  61  21  17  32  54  53  17 | 60  64  58  59  48  58  59  84  61  48  52  60  60  58  58  57  60  60  60  50  46  55  59  94  64  22  19  33  56  57  18 | 60  64  58  58  48  58  59  85  61  48  52  61  58  59  58  60  60  60  50  46  55  50  59  93  64  22  19  34  56  57  18 | 59  65  58  59  48  58  61  81  61  49  52  59  58  59  58  59  60  61  51  46  54  52  59  88  64  24  19  36  57  56  18 | 58  65  48  48  38  48  60  81  62  49  52  59  59  60  59  58  60  60  51  46  54  52  59  89  64  23  20  36  56  57  18 | 54  58  51  55  44  55  55  75  56  45  50  56  53  53  52  55  55  55  46  41  50  47  53  83  56  21  18  32  53  52  17 | 60  62  57  56  45  56  58  71  56  50  50  57  55  55  54  58  59  59  50  43  54  49  56  76  64  20  19  35  51  52  18 | 60  65  59  59  48  59  60  85  60  49  53  61  58  59  58  60  60  60  51  46  55  50  60  94  65  22  19  34  56  57  18 | 58  65  57  58  48  58  60  79  61  48  52  60  58  59  58  59  60  61  50  46  54  52  60  86  64  23  19  36  56  56  19 | 58  65  59  59  47  58  61  78  62  49  52  60  58  59  57  60  61  61  51  46  54  52  59  86  66  22  18  35  55  56  18 |

**Table S2.** Primer sequences and PCR conditions for *OsCHS* cloning

| **Gene** | **Primer Sequence ^a^** | **Annealing**  **Temp. (℃)** |
| --- | --- | --- |
| OsCHS1  (Os01g41834) | 5’-CCGCTAGCATGGCAGCGGAAAATGGCCA-3’  5’-CCGAATTCTTAATTAATGGGCAGCGCGC-3’ | 54 |
| OsCHS2  (Os04g01354) | 5’-GGCATATGGGTAGCCAAGAATACAT-3’  5’-CCGAATTCTCAGACGGCTTGGGCTTGGT-3’ | 54 |
| OsCHS3  (Os04g23940) | 5’-GGCATATGGGATCCTCGGGGGCACC-3’  5’-CCGAATTCTTAATTGCGTGGCATCGCGC-3’ | 54 |
| OsCHS4  (Os05g12180) | 5’-GGCATATGCCTGGAACAGCTACTGC-3’  5’-CCGAATTCTCATGAGAGTGGGTTACGCA-3’ | 54 |
| OsCHS7  (Os05g12240) | 5’-GGCATATGCCTGGAGCAGCTACTGC-3’  5’-CCGAATTCTCAAGAGAGCGGGTTACGCA-3’ | 54 |
| OsCHS8  (Os07g11440) | 5’-GGCATATGGTGACATCAACAGTGAA-3’  5’-CCGAATTCTTACTGCATAATAAGAGGGG-3’ | 54 |
| OsCHS9  (Os07g17010) | 5’-GGCATATGGCACCGACGACGACCAT-3’  5’-CCGAATTCTTAATTCACATGAGAGGTGG-3’ | 54 |
| OsCHS12  (Os07g31770) | 5’-GGCATATGGCACCTGTTCCGGCCAC-3’  5’-CCGAATTCTTAATTTCCCTTGAGGCCGC-3’ | 54 |
| OsCHS15  (Os07g34260) | 5’-GGCATATGGGGAGCACTCCGGCTGC-3’  5’-CCGGATCCCTAATTCTTCTTCCTGCTGT-3’ | 54 |
| OsCHS16  (Os10g07040) | 5’-GGCATATGCCTGGAGCAACTACCGC-3’  5’-CCGAATTCTTAATTTTCCTTCAAACCAC-3’ | 54 |
| OsCHS18  (Os10g08620) | 5’-GGCATATGCCTGGAGCAGCTACCAC-3’  5’-CCGAATTCCTAATTTTGCTTAAGACCAC-3’ | 54 |
| OsCHS22  (Os10g34360) | 5’-GGCATATGGCTGACCTTGGATTCGG-3’  5’-CCGAATTCTCAGTTAATGCCTCGAACTA-3’ | 54 |
| OsCHS24  (Os11g32650) | 5’-GGCATATGGCGGCGGCGGTGACGGT-3’  5’-CCGAATTCTCAGGCGGCGGCGCCGGCGG-3’ | 54 |
| OsCHS25  (Os11g35930) | 5’-GGCATATGGGATCGTCAGGGGCACC-3’  5’-CCGAATTCCTAGCTGTTACGTGGCACTG-3’ | 54 |

^a^ Underlines indicate the restriction sites for the cloning of *OsCHS* into pET28a(+)

**Table S3.** Primer sequences for quantitative real-time PCR analysis

| **Gene** | **Primer Sequence ^a^** | **Annealing**  **Temp. (℃)** |
| --- | --- | --- |
| OsCHS8  (Os07g11440) | 5’- AGGAGGTTAGGAGGATGCAAAG -3’  5’- AGCTTTCACACATCCTCTGGAAC -3’ | 59 |
| OsCHS24  (Os11g32650) | 5’- GGATGTGTGACAAGTCGCAGAT -3’  5’- CGACGACGACGATGTCCTG -3’ | 59 |
