# Supplementary material for: Two Chalcone Synthase Isozymes Participate Redundantly in UV-Induced Sakuranetin Synthesis in Rice
Source: Int J Mol Sci. 2020 May 27;21(11):3777. doi: 10.3390/ijms21113777 (PMC7312121; doi:10.3390/ijms21113777)
Supplement: Supplementary file 1 [file ijms-21-03777-s001.zip › ijms-802367-Supplementary-2/Supplementary figures-R1.pptx]

## Slide 1
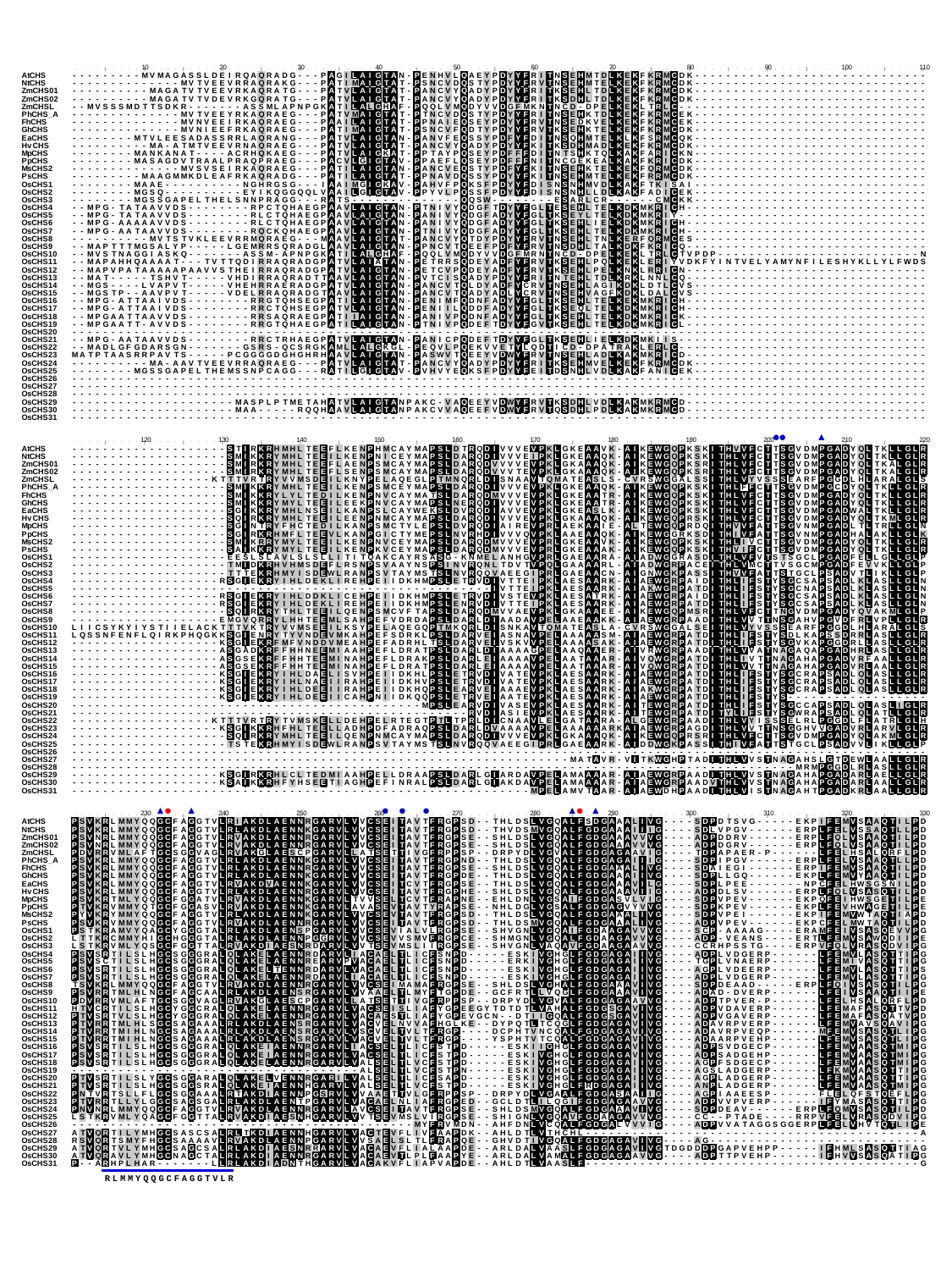

RLMMYQQGCFAGGTVLR

## Slide 2
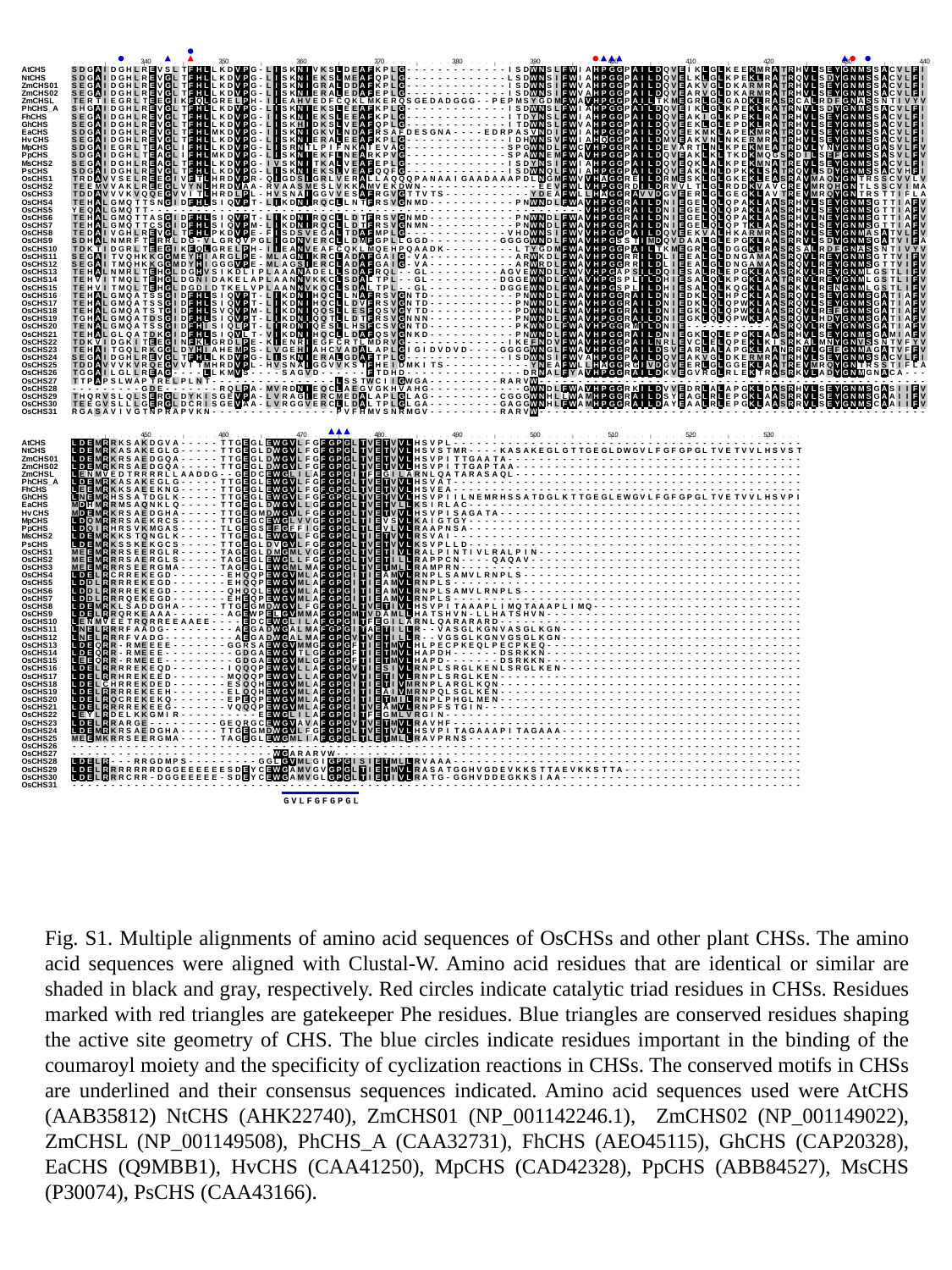

GVLFGFGPGL
Fig. S1. Multiple alignments of amino acid sequences of OsCHSs and other plant CHSs. The amino acid sequences were aligned with Clustal-W. Amino acid residues that are identical or similar are shaded in black and gray, respectively. Red circles indicate catalytic triad residues in CHSs. Residues marked with red triangles are gatekeeper Phe residues. Blue triangles are conserved residues shaping the active site geometry of CHS. The blue circles indicate residues important in the binding of the coumaroyl moiety and the specificity of cyclization reactions in CHSs. The conserved motifs in CHSs are underlined and their consensus sequences indicated. Amino acid sequences used were AtCHS (AAB35812) NtCHS (AHK22740), ZmCHS01 (NP_001142246.1), ZmCHS02 (NP_001149022), ZmCHSL (NP_001149508), PhCHS_A (CAA32731), FhCHS (AEO45115), GhCHS (CAP20328), EaCHS (Q9MBB1), HvCHS (CAA41250), MpCHS (CAD42328), PpCHS (ABB84527), MsCHS (P30074), PsCHS (CAA43166).

## Slide 3
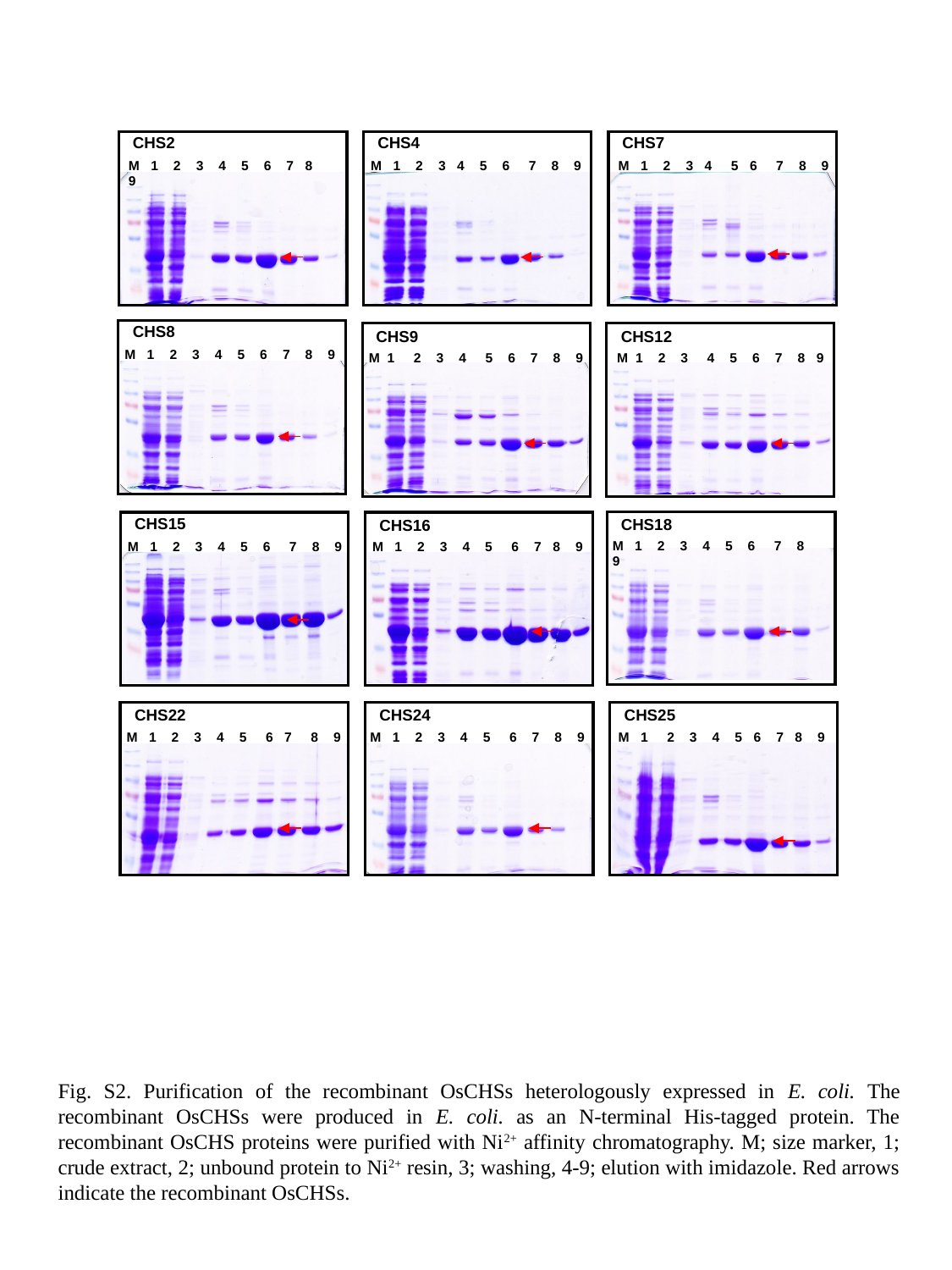

CHS2
CHS4
CHS7
M 1 2 3 4 5 6 7 8 9
M 1 2 3 4 5 6 7 8 9
M 1 2 3 4 5 6 7 8 9
CHS8
CHS9
CHS12
M 1 2 3 4 5 6 7 8 9
M 1 2 3 4 5 6 7 8 9
M 1 2 3 4 5 6 7 8 9
CHS15
CHS18
CHS16
M 1 2 3 4 5 6 7 8 9
M 1 2 3 4 5 6 7 8 9
M 1 2 3 4 5 6 7 8 9
CHS22
CHS24
CHS25
M 1 2 3 4 5 6 7 8 9
M 1 2 3 4 5 6 7 8 9
M 1 2 3 4 5 6 7 8 9
Fig. S2. Purification of the recombinant OsCHSs heterologously expressed in E. coli. The recombinant OsCHSs were produced in E. coli. as an N-terminal His-tagged protein. The recombinant OsCHS proteins were purified with Ni2+ affinity chromatography. M; size marker, 1; crude extract, 2; unbound protein to Ni2+ resin, 3; washing, 4-9; elution with imidazole. Red arrows indicate the recombinant OsCHSs.

## Slide 4
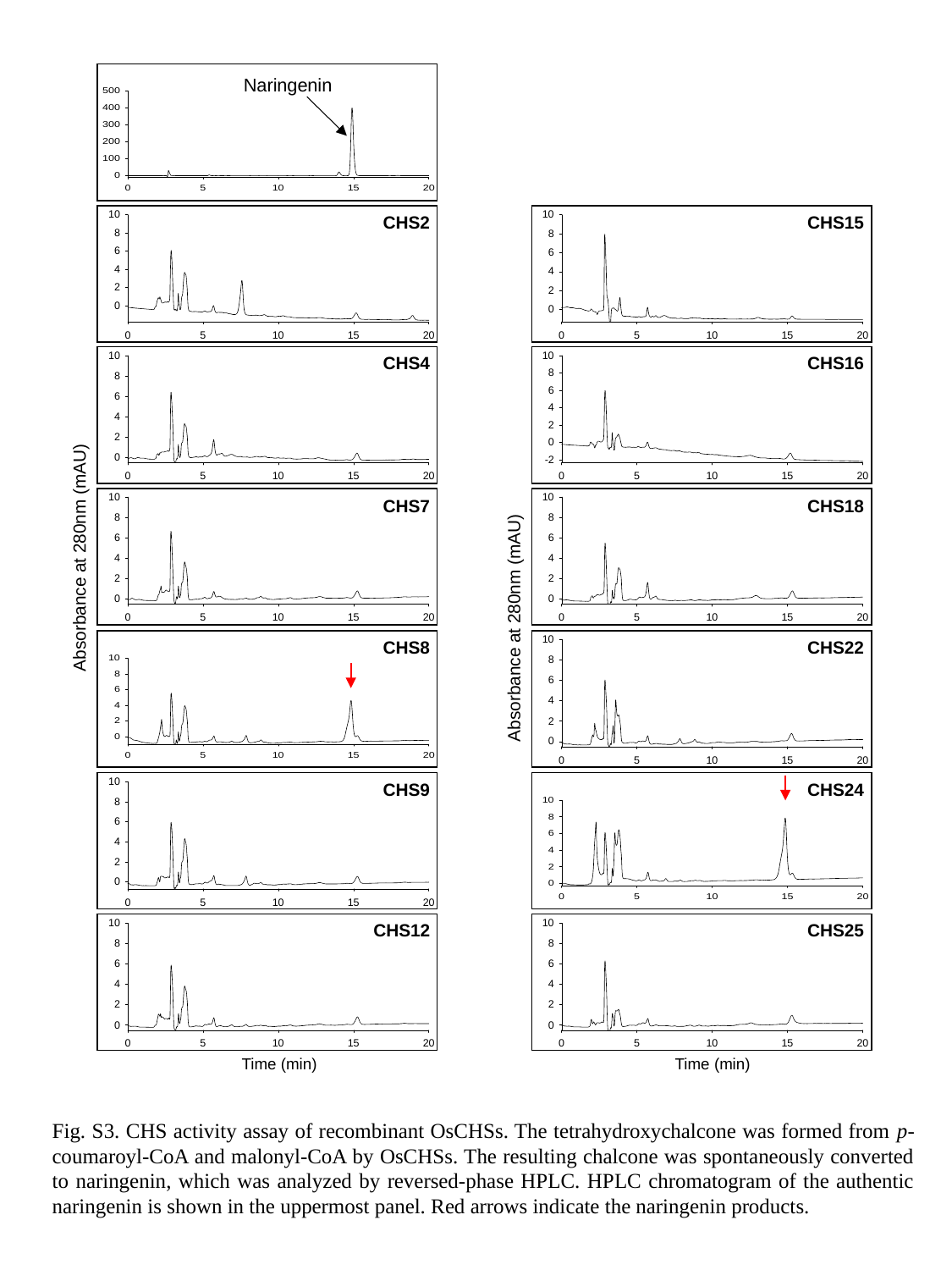

Naringenin
CHS2
CHS15
CHS4
CHS16
CHS7
CHS18
Absorbance at 280nm (mAU)
Absorbance at 280nm (mAU)
CHS8
CHS22
CHS9
CHS24
CHS12
CHS25
Time (min)
Time (min)
Fig. S3. CHS activity assay of recombinant OsCHSs. The tetrahydroxychalcone was formed from p-coumaroyl-CoA and malonyl-CoA by OsCHSs. The resulting chalcone was spontaneously converted to naringenin, which was analyzed by reversed-phase HPLC. HPLC chromatogram of the authentic naringenin is shown in the uppermost panel. Red arrows indicate the naringenin products.

## Slide 5
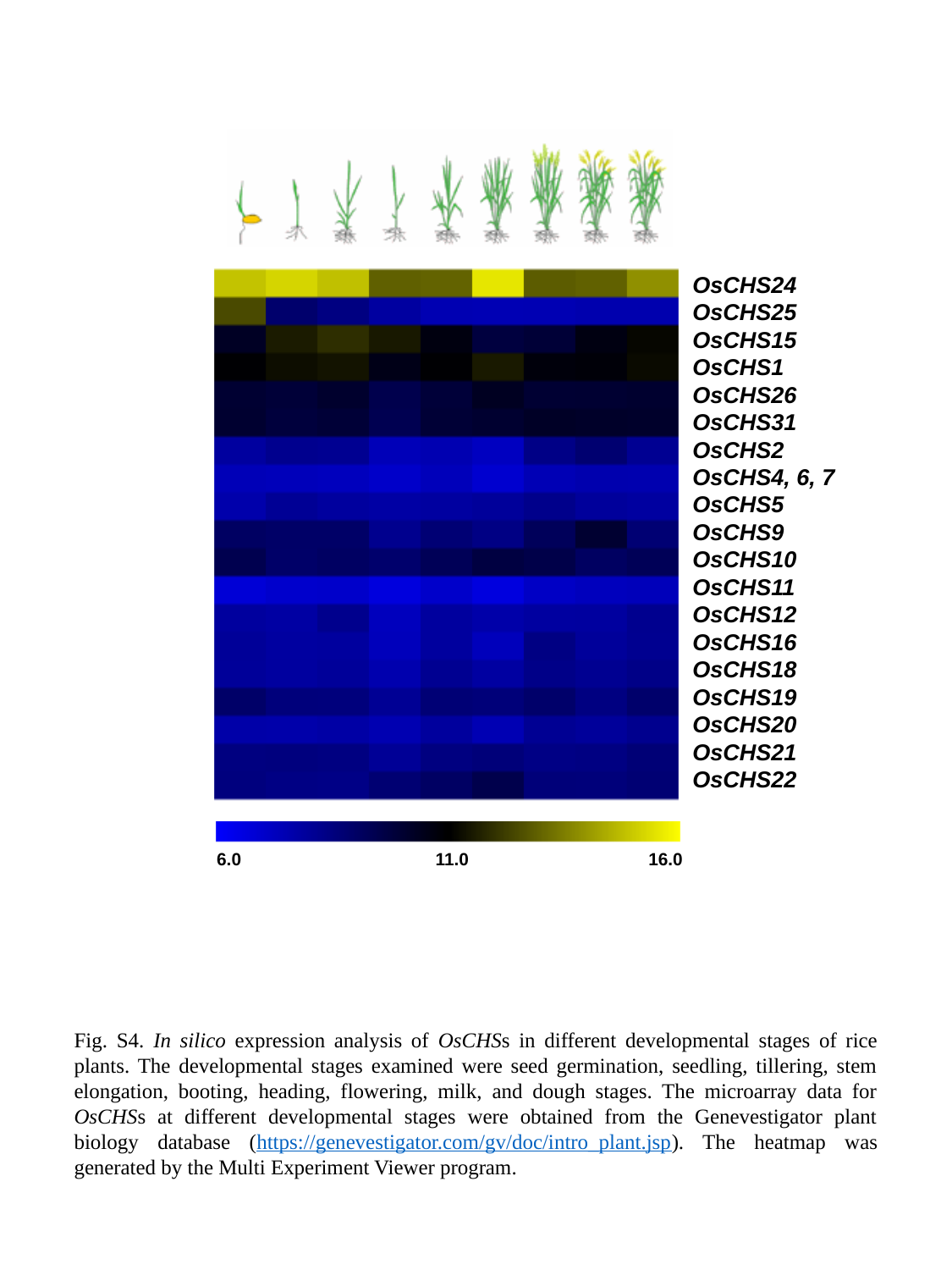

OsCHS24
OsCHS25
OsCHS15
OsCHS1
OsCHS26
OsCHS31
OsCHS2
OsCHS4, 6, 7
OsCHS5
OsCHS9
OsCHS10
OsCHS11
OsCHS12
OsCHS16
OsCHS18
OsCHS19
OsCHS20
OsCHS21
OsCHS22
6.0 11.0 16.0
Fig. S4. In silico expression analysis of OsCHSs in different developmental stages of rice plants. The developmental stages examined were seed germination, seedling, tillering, stem elongation, booting, heading, flowering, milk, and dough stages. The microarray data for OsCHSs at different developmental stages were obtained from the Genevestigator plant biology database (https://genevestigator.com/gv/doc/intro_plant.jsp). The heatmap was generated by the Multi Experiment Viewer program.
